# The role of kidney biopsy in a patient with juvenile SLE with no urine abnormity or no signs of renal failure

**DOI:** 10.1186/1546-0096-9-S1-P255

**Published:** 2011-09-14

**Authors:** M Schüller, J Franova, M Macku

**Affiliations:** 1 Pediatric Department, University Hospital Brno, Czech Republic

## Background

Lupus nephritis belongs to the most serious organ involvements in juvenile systemic lupus erythematosus (SLE) and its adequate treatment is crucial for the prognosis of the disease. The intensity of the treatment should be determined by the kidney biopsy findings scored by the WHO or ISN/RPS classifications. Considering the benefits and risks of the kidney biopsy, some authors have suggested indications for its performance in lupus nephritis based on clinical manifestations comprising significant proteinuria, haematuria with proteinuria, presence of cellular casts and elevated serum creatinine. However, many studies have revealed notable discrepancies between the clinical signs and biopsy findings. Thus, performing kidney biopsy especially in a juvenile SLE patient with no clinical signs of renal disease remains controversial.

## Aim

To emphasize the role of renal biopsy in patients with juvenile SLE with normal clinical kidney manifestations.

## Methods

A case report of a patient diagnosed and treated in a pediatric rheumatology centre in multidisciplinary collaboration.

## Results

A thirteen-year-old boy presented with arthritis and pleural effusion. His laboratory tests showed elevated ESR, anemia, leukopenia, decreased C3 and C4, positive ANA and anti-dsDNA. Despite normal urine sediment, creatinine clearence and no proteinuria, kidney biopsy was performed and revealed diffuse proliferative lupus glomerulonephritis class IV-S(A) according to the ISN/RPS 2003 classification. In the next course Libman-Sacks endocarditis occurred. With diagnose of juvenile SLE with severe kidney involvement the patient was treated with corticosteroids and intravenous cyclophosphamide followed by azathioprine, which was later switched to cyclosporine A, subsequently in combination with mycophenolate mofetil. Despite the serious course of the disease urine analysis and creatinine clearence remained normal for the whole period.

## Conclusions

Based on our experience with a patient with juvenile SLE with severe lupus nephritis despite their normal urine analysis and creatinine level, in accordance with some other authors, we recommend performing kidney biopsy in newly diagnosed juvenile SLE patients unless the risk to benefit ratio of the procedure is very high.

